# Payload Design and Evaluation of *Staphylococcus epidermidis* Adhesion to Nonfouling Polyampholyte Coatings Onboard the International Space Station

**DOI:** 10.3390/molecules30040836

**Published:** 2025-02-11

**Authors:** Adrienne Shea, Kaitlyn Harvey, Ashley Keeley, Hannah Johnson, Niko Hansen, Roslyn McCormack, Kael Stelck, Travis Lindsay, Adriana Bryant, Matthew T. Bernards

**Affiliations:** Department of Chemical and Biological Engineering, University of Idaho, Moscow, ID 83844, USA; shea2697@vandals.uidaho.edu (A.S.);

**Keywords:** *S. epidermidis*, polyampholytes, nonfouling, microgravity, International Space Station

## Abstract

The accumulation of biofilms can potentially be very costly in terms of damage to mechanical systems and health impact on the human body. Space travel, especially long-term space travel, compounds the complications that arise from the accumulation of biofilms because of the lack of access to resources. This study investigates the ability of polyampholyte copolymer thin films to reduce bacteria adhesion in microgravity. Copolymer systems of [2-(acryloyloxy)ethyl] trimethylammonium chloride (TMA) and 2-carboxyethyl acrylate (CAA) and TMA and 3-sulfopropyl methacrylate potassium salt (SA) have previously shown resistance to bacteria adhesion under gravity-impacted conditions. However, their performance under microgravity conditions has never been evaluated. A self-contained payload was designed around multiple constraints to evaluate the ability of the TMA/CAA and TMA/SA thin film coatings to reduce the adhesion and biofilm formation of *Staphylococcus epidermidis* on aluminum test coupons in microgravity in an experiment conducted onboard the International Space Station (ISS). An Earth-based, gravity-impacted study was completed in parallel with the ISS experiment. The samples were then analyzed on the macroscale using photography and the microscale using confocal microscopy imaging to determine biofilm formation and bacteria attachment, respectively. The percentage of each sample covered by bacteria and/or biofilm was characterized and compared amongst the coating types and gravity exposure conditions. The TMA/SA coatings showed the lowest levels of bacteria adhesion and biofilm formation overall. The TMA/CAA coatings showed the largest reduction in bacteria adhesion and biofilm formation when comparing adhesion between the microgravity- and gravity-impacted samples. Therefore, both the copolymers demonstrate promise for bacteria-resistant coatings in microgravity.

## 1. Introduction

Biofilms consist of surface-attached accumulations of bacteria, forming a three-dimensional matrix. The National Institute of Health reported that microbial biofilms have a more than five trillion USD economic impact worldwide, while also causing 80% of human microbial infections [1]. This impact is even more concerning when taken in the context of long-term space travel or planetary colonization, where access to medical care or spare mechanical parts is strictly limited. A trip to Mars alone is estimated to take a minimum of 520 days [2]. When this time duration and distance from assistance is taken into consideration, it is imperative to reduce the occurrence of biofilms within spacecraft to make these missions as successful as possible.

The accumulation of biofilms or biofouling can also be problematic, resulting in damage to mechanical systems. Biofouling causes corrosion and surface degradation to the point of structural or functional failure [2]. The direct consequences of biofilm formation within the water reclamation systems of spacecraft include blocked or damaged filters, leading to a deterioration in water quality [3]. For example, the system for recycling atmospheric condensates on the Mir space station had recurring malfunctions, which were attributed to biofilms upon analysis after return [3]. On top of the recurring issues with the water recycling system, the Soviet Union’s Salyut 6, Salyut 7, and Mir also experienced technical difficulties attributed to microbial contamination in multiple additional systems [3]. The International Space Station (ISS) has also suffered from the contamination of their water recovery system [4]. In 2010, part of the water reclamation system on board the ISS was replaced due to increased pressure from biofilm fouling [5].

Beyond the mechanical impacts, biofilms can also have significant impacts on the health of astronauts. A previous investigation has shown that there are changes in gastrointestinal, nasal, and respiratory bacterial flora due to a moderate reduction in immune system competence, leaving astronauts at increased risk of infection [6]. Some studies have also shown that spaceflight increases bacterial proliferation and virulence, while also requiring a greater concentration of antibiotics to inhibit their growth [7]. During early habitation on the ISS, bacterial characterization was performed that isolated sixty-three bacteria strains. This included twelve bacteria strains that were isolated from the ISS water system and thirty bacteria colonies that were isolated from preflight and flight surfaces, with two of the most common isolates being strains of *Staphylococcus* [8]. Knowing the prevalence biofilms have on the ISS and the potential impact on the crew that inhabit it, there is a need for additional controls to prevent biofilm formation.

The current method for controlling biofilm formation on common surfaces within the ISS includes the weekly use of antimicrobial wipes. These are used in addition to pre-flight sterilization and astronaut quarantine. All these methods have intrinsic merit, but even with these current systems in place, bacteria isolates are still routinely detected on common surfaces within the ISS. The bacteria isolates are also known to be strong biofilm formers and present removal challenges due to increased resistance to disinfectants and other antimicrobials under microgravity conditions [9]. Surface conditioning also results in recurrence that is almost impossible to prevent [10]. Compounded by the fact that these methods require additional weight and astronaut time, which are both at a premium in space, the prevention of biofilm formation rather than treatment is the desired outcome.

**Figure 1:**
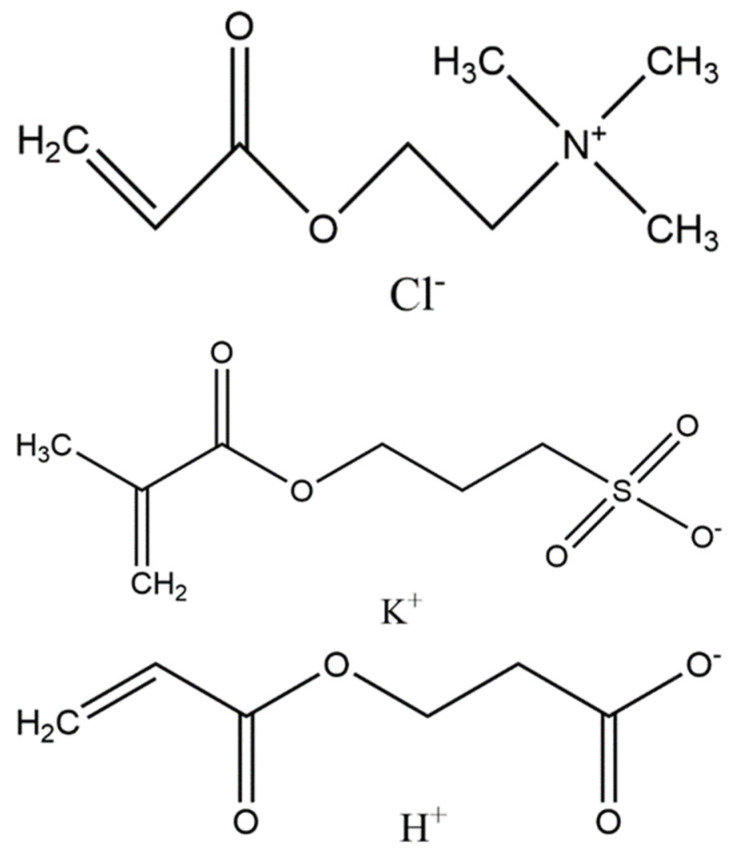
Monomer structures for [2-(acryloyloxy)ethyl] trimethylammonium chloride (TMA) (**top**), 3-sulfopropyl methacrylate potassium salt (SA) (**middle**), and 2-carboxyethyl acrylate (CAA) (**bottom**) used to form polyampholyte nonfouling coatings.

One promising approach for preventing biofilm formation is the utilization of nonfouling polymer coatings. A nonfouling polymer is defined as one that has less than 5 ng/cm^2^ of nonspecific protein adsorption from complex media [11]. These types of polymers have also demonstrated resistance to bacteria adhesion in gravity-impacted investigations [12,13,14,15]. The best-performing nonfouling polymers are based on zwitterionic chemistries, which consist of an equal number of anionic and cationic groups. Polyampholyte polymers are a subset of zwitterionic polymers that are based on mixtures of charged monomers subunits [16]. Polyampholytes have also demonstrated resistance to bacteria adhesion [12,17]. In one recent study, a [2-(acryloyloxy)ethyl] trimethylammonium chloride (TMA) and 2-carboxyethyl acrylate (CAA) (TMA/CAA) polyampholyte copolymer demonstrated the broadest resistance to bacteria adhesion and growth upon exposure to bacteria sources in a citizen science-based investigation [12]. Bacteria resistance was compared to that of other zwitterionic polymers, including sulfobetaine methacrylate and a TMA and 3-sulfopropyl methacrylate potassium salt (SA) (TMA/SA) polyampholyte copolymer. The TMA/SA copolymer also outperformed the sulfobetaine-based polymer. As a result, these two polyampholyte copolymers were evaluated in this investigation (Figure 1).

In this study, a payload was designed to test the efficacy of the nonfouling polyampholyte polymers in reducing bacterial adhesion to aluminum surfaces under microgravity conditions. This self-contained payload was designed to operate autonomously upon power up onboard the ISS. Only a limited number of experiments have evaluated bacterial biofilm formation in microgravity without crew intervention, and this prior work was completed with different design constraints [18]. The design constraints for this study included the requirement of two independent levels of containment; the isolation of the bacteria until power up in microgravity; and size, weight, and power restrictions. Aluminum 6061 coupons were coated with one of the two polyampholyte copolymers (TMA/CAA or TMA/SA), placed inside the chamber, and then exposed to *Staphylococcus epidermidis* once onboard the ISS alongside uncoated aluminum control coupons. An Earth-based, gravity-impacted study was completed in parallel with the ISS experiment. *S. epidermidis* was chosen as the bacterium strain to be studied for this project because this strain is developing antibiotic-resistant properties [19], while also being prevalent on common contact surfaces onboard the ISS. *S. epidermis* has been isolated from multiple control panels, as well as a treadmill, and determined to be consistent with the isolates characterized from preflight surfaces [8]. Both the experimental systems (the gravity-impacted “Earth box” and the ISS “Space Box”) were loaded with three samples each of TMA/CAA copolymer-coated aluminum, TMA/SA copolymer-coated aluminum, and uncoated aluminum, which were randomly distributed in a 3 × 3 square. The bacterium was exposed to the coupons and allowed to form biofilms for identical time periods before qualitative and quantitative analyses were performed on them to determine bacteria attachment and biofilm formation. The results suggest that the TMA/SA coating led to the lowest levels of bacteria adhesion and biofilm formation, while the TMA/CAA coating led to the greatest reduction in biofilm formation between the ISS and the Earth-based control. However, there is a significant opportunity to improve the overall coating uniformity and the performance.

## 2. Discussion and Results

In the first stage, an experimental platform was designed to allow for the direct assessment of the impact of microgravity conditions on the bacteria-resistance properties of the nonfouling polymers. This platform was designed to fit within a 1.5U Nanoracks housing and to operate with power and weight limitations. Further, the crew operational requirements onboard the ISS were limited to the connection of the system to power. The final design of the system and the electrical components are shown and described in more detail in the Section 3. The objective was to evaluate the performance of polyampholyte coatings under microgravity conditions, so it was important to ensure that the bacteria were not introduced until the payload was installed onboard the ISS. This was completed by transporting lyophilized *S. epidermidis* enclosed within the bacteria introduction pistons and introducing them to the growth media upon powerup onboard the ISS. Further, an identical experimental system was built and operated on Earth as a gravity-impacted control.

**Figure 2:**
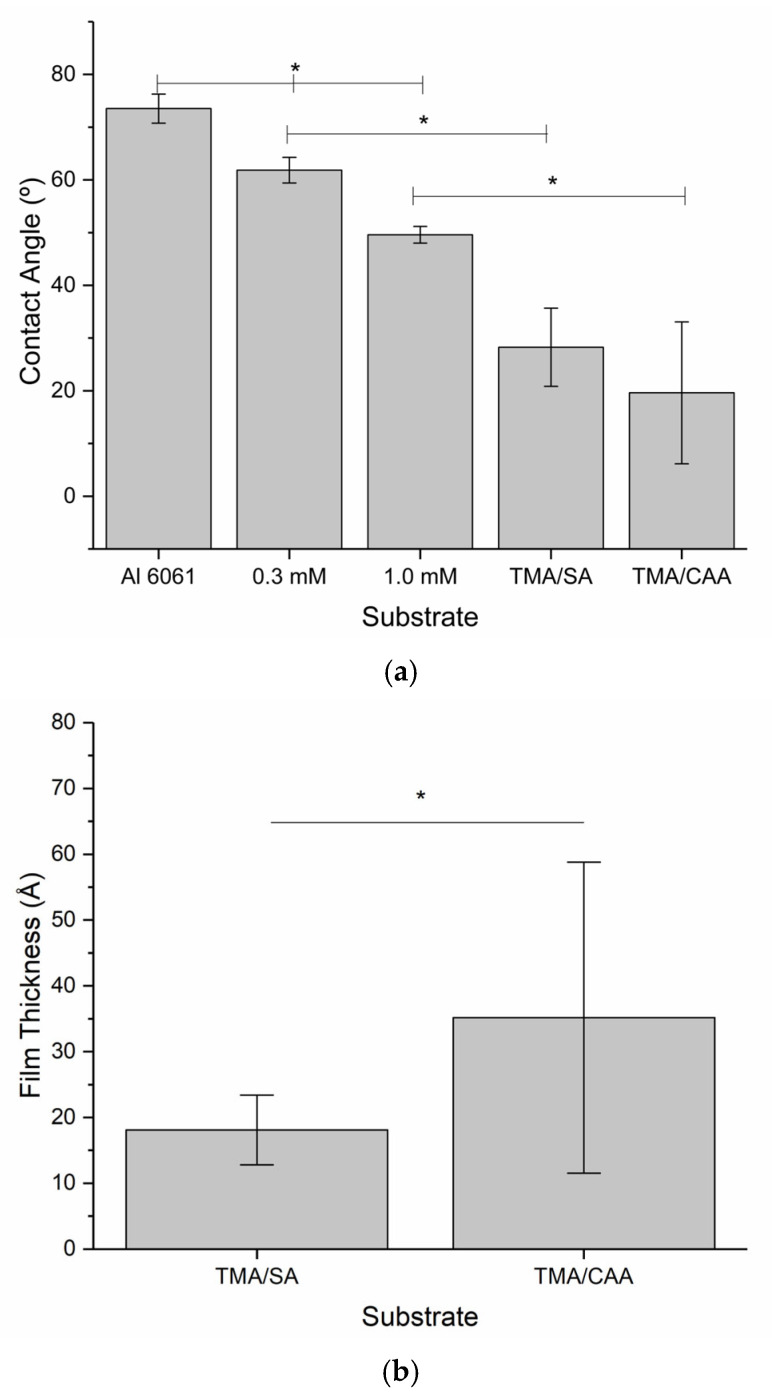
Mean ± SD of the (**a**) contact angle for substrates before and after thin film hydrogel coating and (**b**) film thickness measurements for the thin film hydrogels. * indicates a statistically significant difference between indicated samples at a 95% confidence level (*p* < 0.05).

Due to a compressed project timeline (<1 year from project kick-off to launch), detailed characterization of the thin film coatings was not completed until after the payload had returned from the ISS, while bacteria adhesion and biofilm evaluations were on-going. The thin film coatings were first evaluated using contact angle goniometry to confirm the presence of the thin film hydrogels. The polyampholyte coatings were expected to be significantly more hydrophilic than the bare aluminum substrates, and the contact angle measurements shown in Figure 2a confirm this. Additionally, the thin film hydrogel coatings are significantly more hydrophilic than the surface initiator-coated aluminum substrates, indicating that the polymers are responsible for the enhancement of surface wettability. However, the contact angle measurements for the polyampholyte coatings also demonstrate the largest error, indicating that the coatings are not as uniform as the bare aluminum and initiator-coated aluminum.

Ellipsometry measurements were used to assess the thickness of the thin film coatings used in this study, and the results are shown in Figure 2b. This analysis indicated that the coatings were in the order of 20–40-angstroms-thick, with significant variations in the values obtained for the TMA/CAA coatings. When these results are coupled with the contact angle measurements, it is apparent that the drop coating techniques selected for this study lead to highly nonuniform thin film polymer coatings. This was confirmed when the surface roughness was evaluated using atomic force microscopy, which also indicated a highly nonuniform surface topology. Coating thickness is known to impact the performance of nonfouling polyampholyte polymer coatings [20]. However, Konradi et al. found that more significant impacts are caused by the patchiness of film coverages, especially in the lower extremes of film thickness [21]. Imperfect films still have the capacity to reduce bacterial adhesion, but they are unable to completely prevent it because imperfections in the coatings create points of bacterial adhesion, leading to biofilm formation [21]. Therefore, it is reasonable to assume that this impacted the bacterial adhesion results discussed below.

Polyampholytes have demonstrated resistance to bacteria adhesion in prior gravity-impacted investigations [12,15,22]. It was hypothesized that the bacteria-resistant performance would be enhanced under microgravity conditions due to the reduction in the gravity-induced compression of the coating and bacteria settling. The performance of the polyampholyte thin film coatings were evaluated following 32 days of *S. epidermidis* exposure in microgravity and 3 days of exposure in gravity, while the payload was in transit following the return to Earth. The levels of bacteria adhesion were evaluated from both macroscopic and microscopic perspectives using photography and confocal microscopy, respectively.

**Figure 3:**
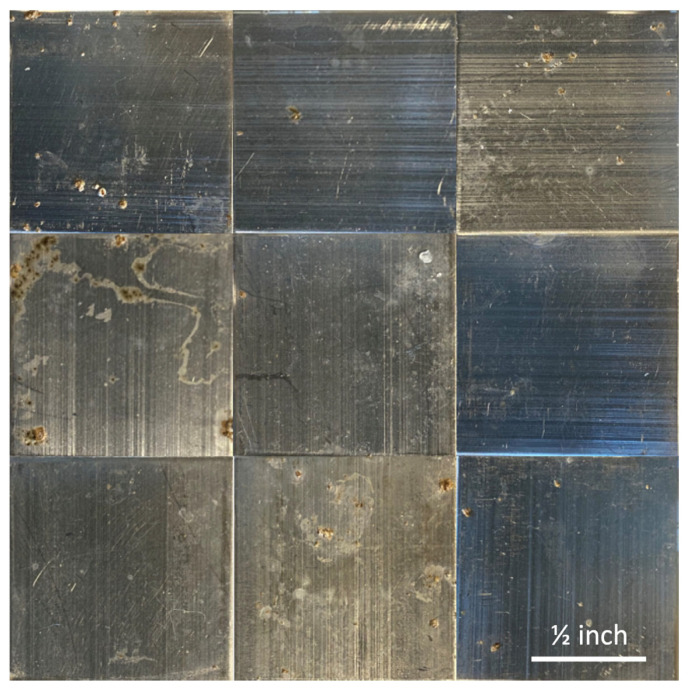
Photographs of the ISS samples following exposure to bacteria, including the uncoated aluminum 6061 controls (**top row**), the TMA/CAA thin film hydrogel (**middle row**), and the TMA/SA thin film hydrogel (**bottom row**). The scale bar in the bottom right image represents 0.5 inches and is representative of all the images.

**Figure 4:**
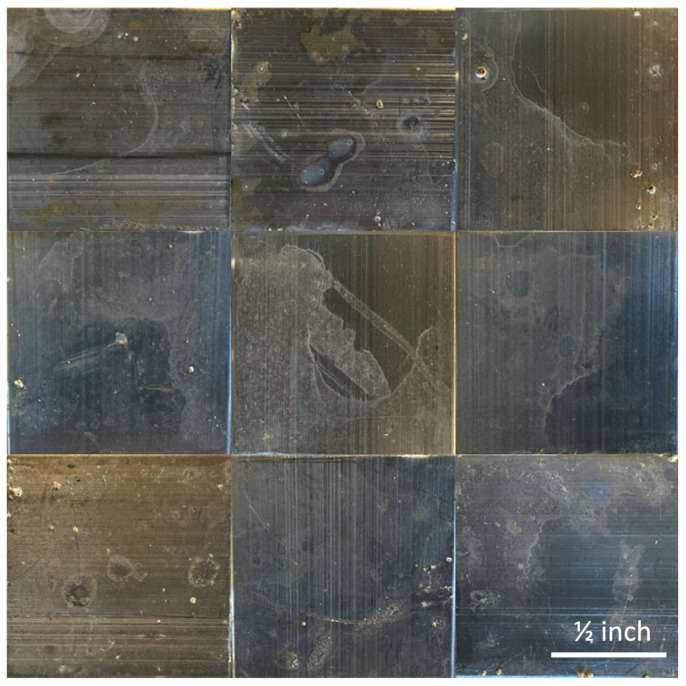
Photographs of the Earth-based samples following exposure to bacteria, including the uncoated aluminum 6061 controls (**top row**), the TMA/CAA thin film hydrogel (**middle row**), and the TMA/SA thin film hydrogel (**bottom row**). The scale bar in the bottom right image represents 0.5 inches and is representative of all the images.

Figure 3 shows photographs captured of the samples exposed to *S. epidermidis* onboard the ISS. It is clear there is minimal biofilm formation on all the samples, including both the uncoated controls and the polyampholyte-coated samples. Similarly, Figure 4 shows photographs captured of the control samples exposed to *S. epidermidis* on Earth. It is clear that there are significantly greater levels of biofilm formation across all the samples relative to the ISS samples.

**Figure 5:**
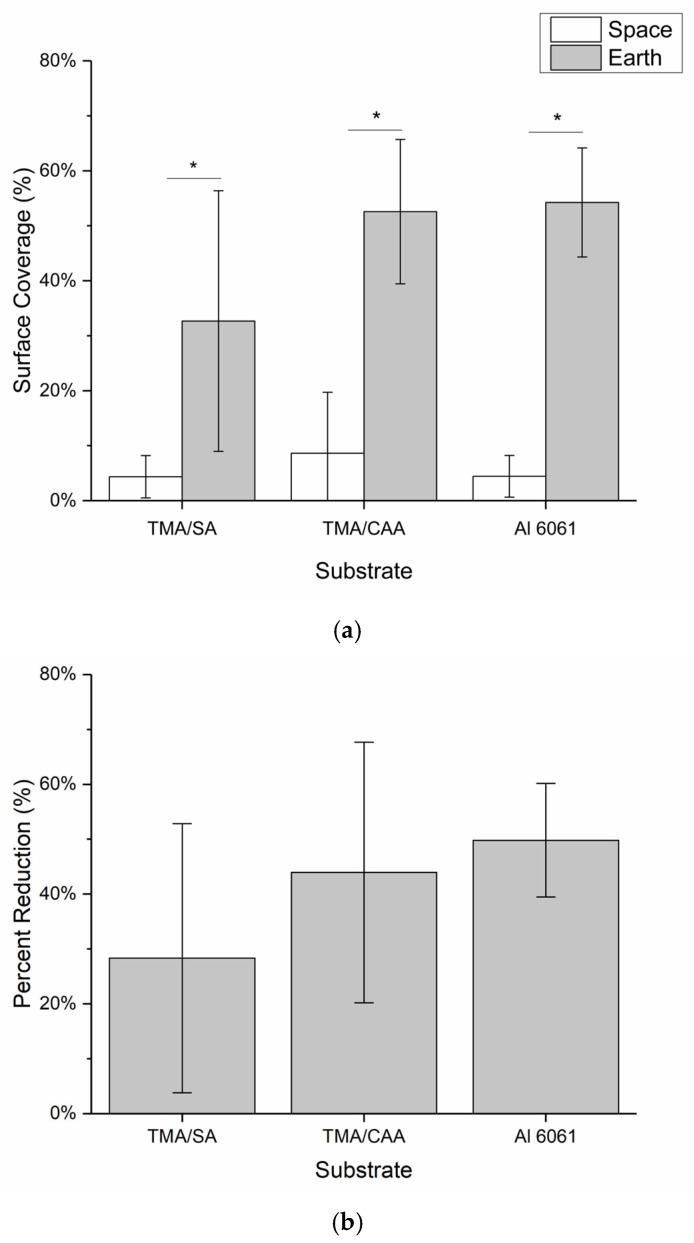
The mean ± SD of the (**a**) biofilm surface coverage of the samples based on the analysis of the photographs captured of each sample and (**b**) percent reduction in the biofilm surface coverage for the samples on the ISS relative to that of their Earth-based controls. * indicates a statistically significant difference between the indicated samples at a 95% confidence level (*p* < 0.05).

These images were then evaluated by two independent, blinded researchers to identify all the areas where biofilm was potentially present in each of the images using ImageJ. This evaluation was then used to calculate the maximum overall surface coverage of biofilm for each substrate coating, and the results are shown in Figure 5a. The results in Figure 5a indicate that the ISS samples had overall average biofilm coverages of <10%, with the TMA/SA and uncoated aluminum samples performing the best (not statistically different). All three substrates performed statistically better than their Earth-based counterparts, which had biofilm surface coverage ranging from 30 to 55%. The TMA/SA polyampholyte coating also performed the best under the gravity-impacted conditions, but again the results are not statistically significant. In Figure 5b, the overall reduction in biofilm surface coverage is presented based on the relative biofilm surface coverage between the two systems. It is seen that microgravity reduced the overall biofilm surface coverage by 30–50%, with the TMA/CAA and uncoated aluminum substrates showing the greatest reductions. The results shown in Figure 5 are acknowledged to have large standard deviations. These are attributed to a combination of nonuniform surface coating as discussed above, along with the limited number of samples that were evaluated (n = 3) given the payload constraints.

It is important to acknowledge that the macroscale biofilm results represent the maximum possible surface coverage, but likely provide an overestimate of actual surface coverage. There is the possibility that some of the areas identified as bacteria biofilm could be salt deposits, paraformaldehyde residue, or other surface debris from incomplete rinsing. However, during image analysis, all of these potential surface contaminants were identified as biofilm to ensure that the results represent the worst-case scenario. For this reason, bacterial staining and confocal microscopy imaging were also completed to isolate the bacteria and provide a more accurate quantification of the bacteria adhesion levels.

**Figure 6:**
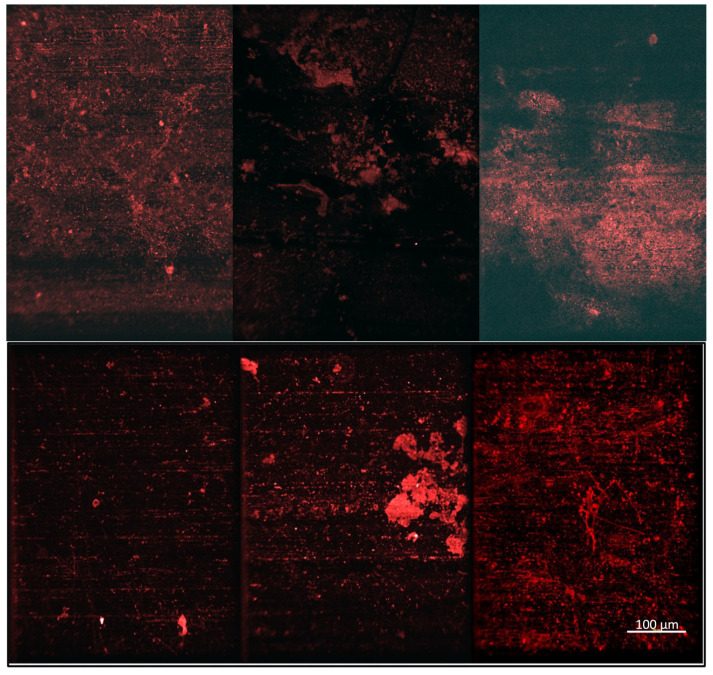
Confocal microscopy images of the Earth-based samples (**top row**) and the ISS-based samples (**bottom row**) following exposure to bacteria, including the uncoated aluminum 6061 controls (**left**), the TMA/CAA thin film hydrogel (**middle**), and the TMA/SA thin film hydrogel (**right**). The scale bar in the bottom right image represents 100 µm and is representative of all the images.

The samples were stained and subjected to confocal microscopy to further evaluate the bacteria adhesion levels for the coated and uncoated substrates at the microscale. Figure 6 shows representative confocal microscopy images of each of the ISS and Earth-based samples. In these images, it can be seen that there is significant background fluorescence caused by striations on the aluminum substrates. ImageJ was used to filter out as much of this as possible before overall fluorescence was quantified for multiple images of each substrate. However, the background fluorescence could not be completely removed without losing the signal from the stained bacteria, so the quantified results were evaluated relative to each other and not as an absolute surface coverage value.

**Figure 7:**
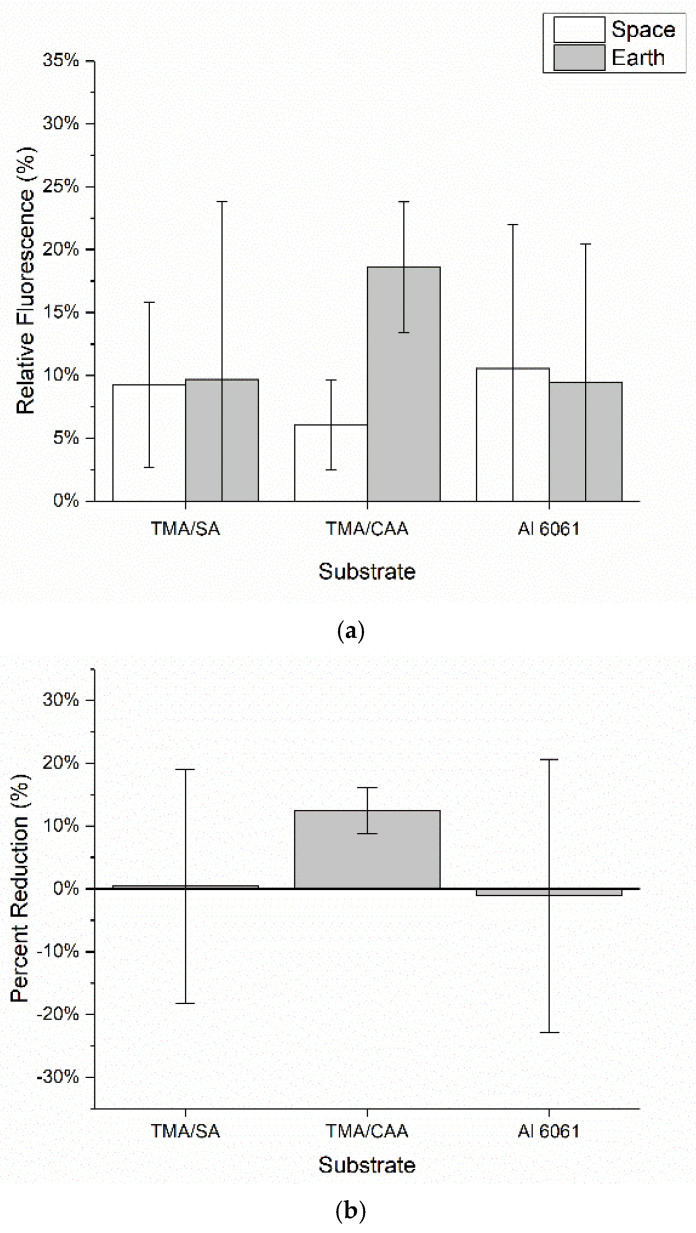
The mean ± SD of the (**a**) overall fluorescence surface coverage of the samples based on the analysis of replicate confocal microscopy images captured of each sample and (**b**) percent reduction in the overall fluorescence surface coverage for the samples on the ISS relative to that on their Earth-based controls.

Figure 7 shows the relative levels of bacteria adhesion on the coated and uncoated substrates following exposure to bacteria on both the ISS and Earth. The relative levels of bacteria adhesion on both the TMA/SA and uncoated aluminum substrates are nearly identical for both the ISS and Earth samples. This suggests the presence of the TMA/SA coating did not have an impact on the overall levels of bacteria adhesion. In contrast, the TMA/CAA coating did demonstrate a reduction in bacteria adhesion in microgravity when compared to that on the gravity-impacted samples. However, the Earth-based TMA/CAA coatings also showed the greatest levels of bacteria adhesion. The TMA/CAA-coated samples were the only ones that demonstrated a reduction in relative fluorescence between their ISS and Earth samples (Figure 7b). As before, there was also significant image-to-image fluorescent signal variability. When this was coupled with background interference, it led to relatively large standard deviations, preventing the results from being statistically different. Despite this, there is still valuable information to be gleaned from the results, especially given the limited number of autonomous ISS payloads that have been designed to evaluate biofilm reduction strategies [18].

Despite the fact that spaceflight has been shown to increase bacteria proliferation and virulence [7], this was not seen in this investigation. At the macroscale, there was a significant reduction in the amount of biofilm formation across the substrates. At the microscale, there were identical or reduced levels of bacteria adhesion depending on the substrate. Broadly, the results suggest that the TMA/SA polyampholyte coating shows the most promise because it had the lowest relative levels of bacteria adhesion for both the ISS- and Earth-based conditions. The TMA/CAA-coated substrates had the greatest reduction in bacteria adhesion on the ISS relative to that on their Earth-based controls. This suggests that these coatings were most impacted by microgravity, and this may be related to the greater thickness of these coatings compared to the TMA/SA thin films (Figure 2b). However, all the results were impacted by nonuniform coatings that resulted from the naturally occurring striations in the aluminum substrates and the drop coating approach used to apply the thin films. These two concerns are being directly addressed in the next iteration of this investigation, which is currently being designed and conducted with the use of polished metal substrates and alternative coating procedures.

## 3. Materials and Methods

### 3.1. Materials

Ethanol (99%) was purchased from Greenfield Global (Toronto, ON, Canada). Ethylene glycol (99.8% anhydrous), sodium hydroxide (NaOH; ≥97%), triethylene glycol dimethacrylate (TEGDMA; 95%), TMA (80% weight in water), CAA (≥99%), SA (≥98%), ammonium metabisulfite (SMS; ≥99%), ammonium persulfate (APS; ≥98%), phosphate buffered saline (PBS), dimethyl sulfoxide (DMSO), and a Baclight red bacterial viability kit were purchased from Sigma-Aldrich (St. Louis, MO, USA). 3-(Trimethoxysilyl) propyl 2-bromo-2-methylpropanoate (92%) was purchased from Gelest (Morrisville, PA, USA). All chemicals were ACS-grade or better and used as received. *Staphylococcus epidermidis* was purchased from the American Type Culture Collection (ATCC 14990, Manassas, VA, USA). BD Difco nutrient broth and bacteriological agar were purchased from Fisher Scientific (Waltham, MA, USA). Aluminum 6061 was purchased from McMaster-Carr (Elmhurst, IL, USA) and cut into 2.54 cm (1 inch) squares (6.45 cm^2^).

### 3.2. Payload Components

White Delrin acetyl resin, fastener hardware, high-temperature soft silicone O-rings and 1.27 cm (0.5 inch) compression springs were purchased from McMaster-Carr (Elmhurst, IL, USA). Linear actuators were purchased from Actuonix (Saanichton, BC, Canada). Two Arduino Teensy microcontrollers, silicone wrapped wiring, and other electrical components were purchased from Adafruit (New York, NY, USA). Gikfun DC microfluidic pumps were purchased from Amazon (Seattle, WA, USA), and Loctite Stik’n Seal Extreme Conditions and 3M double-sided mounting tape were purchased from local retailers.

### 3.3. Payload Design and Assembly

**Figure 8:**
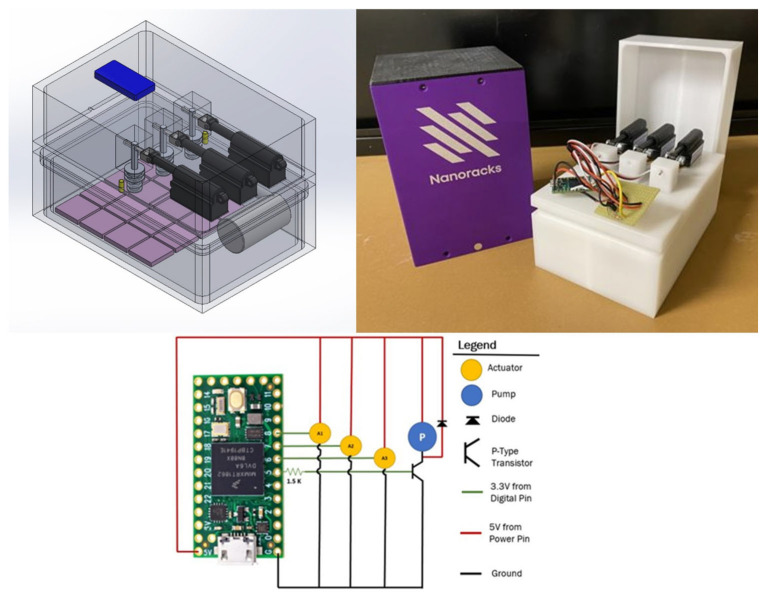
Images of the experimental payload design (**top left**), the experimental build (**top right**), and the electrical circuit diagram (**bottom**). In the payload design, the light purple squares represent the experimental substrates, the blue rectangle represents the Arduino Teensy, and the black objects are the Actuonix linear actuators.

The experimental payload was designed to fit within a 1.5U Nanoracks housing and to operate under the power and operating constraints imposed by this project. The wet space of the payload was designed as a set of two nested Delrin boxes to provide two independent levels of containment, as required for biosafety level 1 bacteria onboard the ISS. The payload’s dry space was a single Delrin box that housed the electrical components. The wet and dry spaces were divided by two wet space containment lids. These lids also included the bacteria introduction devices (BIDs) which passed through the innermost containment lid. The BIDs were composed of spring-loaded pistons machined with a space where the lyophilized bacteria were loaded prior to launch. The pistons held containment with O-ring gaskets that sealed them to the lids. All the payload components were machined from blocks of Delrin to eliminate leak points. As a result, the only sealing requirement was gluing the wet space lids to their respective containment boxes. This was done with Loctite “Stik’n Seal Extreme Conditions”. The payload design is shown in Figure 8 (top left), and the actual experimental build is shown in Figure 8 (top right).

The electrical system for the payload was designed with operating constraints of 5 volts and 400 mA. The overall system was composed of an Arduino Teensy, three linear actuators, and a microfluidic pump, as shown in the wiring diagram in Figure 8. The system was controlled with an open-source Arduino platform and PJRC Teensy 4.0. Teensy required 136 mA of current and 0.680 watts during operation. The code was pre-loaded onto the Arduino and activated upon power up onboard the ISS. Each of the Actuonix linear actuators was retracted in series, drawing 194 mA and requiring 0.971 watts each. Finally, the microfluidic pump was activated for 10 s every 5 min to stir the growth media. The pump drew 283 mA of current and required 1.416 watts of power.

Just prior to launch, one unit of lyophilized *S. epidermidis* (ATCC #14990) was divided evenly and placed in the three BID pistons. The pistons were pressed closed and held in place with their respective actuator pins. The actuators were then glued in place to the dry side of the containment lid with Loctite “Stik’n Seal Extreme Conditions”. In parallel, 500 mL of BD Difco nutrient broth was prepared and sterilized. The polymer-coated substrates were removed from PBS and mounted within the payload’s wet space using 3M mounting tape, and the space was subsequently filled with sterile BD Difco nutrient broth. Following this, the lids were sealed to the two containment levels using Loctite “Stik’n Seal Extreme Conditions”. After the Loctite was cured, the payload was loaded into the Nanoracks housing and handed over for flight.

### 3.4. Thin Film Synthesis

The aluminum coupons were washed in both water and ethanol, and then cleaned for twenty minutes in a Jelight UV/ozone cleaner (Model 342, Irvine, CA, USA). The coupons were then placed in a solution of 3-(trimethoxysilyl) propyl 2-bromo-2-methylpropanoate surface initiator overnight. Following this, the samples were removed from solution, rinsed with ethanol, and air-dried prior to thin film hydrogel synthesis. The TMA/CAA thin films were prepared on substrates exposed to 0.3 mM initiator solution, and the TMA/SA thin films were prepared on substrates exposed to 1.0 mM initiator solution [12]. TMA and CAA or TMA and SA (4 mmol of each monomer) were combined in a solvent solution composed of a volume ratio of 1.5 parts ethylene glycol:1 part ethanol:1.5 parts 6.7 M NaOH. The solution was mixed before adding 0.304 mmol of TEGDMA. The polymerization reaction was initiated by the addition of 32 µL of 40 weight percent APS in ultrapure water and 32 µL of 15 weight percent SMS in ultrapure water. The solution was mixed for 30 s, and then 75 µL of solution was pipetted onto the center of each aluminum coupon. A piece of parafilm was then placed over the top of the coupon to spread the solution, and the samples were placed in a 60 °C oven for one hour. Following the oven, the samples were promptly removed, the parafilm was removed, and the samples were soaked in PBS until use.

### 3.5. Thin Film Characterization

Contact angle measurements were taken in triplicate for the aluminum coupons before and after soaking in the initiator solution, as well as after thin film hydrogel formation (n = 9). All the contact angle measurements were taken using a Ramé-Hart Model 250 standard contact angle goniometer utilizing DROPimage Advanced software (version 3.23.08.0) by placing a 5 µL droplet of ultrapure water onto the samples. Prior to measurement, the samples were removed from PBS buffer and patted dry.

The hydrogel coating thickness was estimated with ellipsometry. A J.A. Woollam M-2000 ellipsometer was used, and ten individual samples of both TMA/CAA-coated aluminum and TMA/SA-coated aluminum were analyzed (n = 10). All the ellipsometry measurements were taken at the Idaho Microfabrication Laboratory at Boise State University (Boise, ID, USA). Prior to measurement, the samples were removed from PBS buffer, rinsed with deionized water, and blown dry with nitrogen.

### 3.6. International Space Station Operations

The experimental payload was launched on 21 December 2021 on Crew Resupply Mission 24 (CRS-24). The payload was attached to power on 23 December 2021, where it ran uninterrupted until it was powered down on 17 January 2022. The payload remained in microgravity until it returned to Earth on 24 January 2022. Upon return, the payload was sent overnight to the University of Idaho, and the bacteria were fixed with 4% paraformaldehyde on 27 January 2022.

### 3.7. Biofilm Evaluation

Following fixation and payload opening, photographic images were immediately captured of all the nine coupons in both the microgravity payload, as well as the Earth-based control (n = 3). The captured images were analyzed by two independent individuals, with their sample identity blinded, to determine the percent surface coverage of biofilm using ImageJ software (Version 1.54g). This was accomplished by highlighting all the areas of each image where biofilm was observed and summing the surface coverage of biofilm relative to the total area.

Following the capture of photographs, the bacteria were then stained using a live/dead BacLight viability kit following the manufacturer’s protocols. While all the bacteria were already dead from the fixation process, the staining allowed for the capture of confocal microscopy images for each of the substrates. Confocal microscopy images were captured with a Nikon spinning disk confocal microscope at a wavelength of 561 nm. Five images were taken of each sample (3 samples; n = 15). The image locations were consistent across the samples as follows: one near each corner and one centrally located on the sample with no overlap in the field of view. The images were then processed using ImageJ to determine the surface coverage of bacteria based on image fluorescence.

### 3.8. Data Analysis

The numbers of independent samples per group and measurements per sample are detailed above. The data were compiled, and the average and standard deviation (SD) of the data sets are shown throughout the paper. Statistical analysis was conducted using OriginPro 2017 using one-way analysis of variance (ANOVA) with a Tukey post-hoc test. The data sets were deemed to be statistically different at a 95% confidence level (*p* < 0.05).

## 4. Conclusions

In this investigation, a 1.5U experimental payload was designed and built to evaluate the ability of thin film polyampholyte coatings to prevent *S. epidermidis* adhesion to aluminum substrates under microgravity conditions. This payload was sent to the ISS on CRS-24, and the coated and uncoated substrates were exposed to a bacterium for 32 days in microgravity. The ability of the TMA/SA and TMA/CAA polyampholyte thin films to prevent bacteria adhesion and biofilm formation were evaluated using photography and confocal microscopy to determine the macroscale and microscale levels of bacteria attachment, respectively. The TMA/SA coatings showed the lowest levels of bacteria adhesion and biofilm formation. At the same time, the TMA/CAA coatings showed the largest reduction in bacteria adhesion and biofilm formation when comparing adhesion between the ISS and Earth-based samples. While these results are promising, the results were not statistically significant due to the significant sample-to-sample variation caused by the nonuniform coatings. Therefore, this experiment is being redesigned, and the polyampholyte performance will be re-evaluated in the near future.

## Data Availability

All data evaluated and presented in this manuscript are available from the corresponding author upon reasonable request.
